# Subarachnoid hemorrhage: New insights on pathogenesis

**DOI:** 10.3389/fstro.2023.1110506

**Published:** 2023-02-06

**Authors:** Divine C. Nwafor, Allison L. Brichacek, Michael S. Rallo, Nina Bidwai, Robert A. Marsh

**Affiliations:** ^1^Department of Neuroscience, School of Medicine, West Virginia University Health Science Center, Morgantown, WV, United States; ^2^Rockefeller Neuroscience Institute, West Virginia University, Morgantown, WV, United States; ^3^Department of Neurosurgery, Rockefeller Neuroscience Institute, West Virginia University, Morgantown, WV, United States; ^4^Department of Microbiology, Immunology, and Cell Biology, School of Medicine, West Virginia University Health Science Center, Morgantown, WV, United States; ^5^Department of Neurosurgery, Rutgers-Robert Wood Johnson Medical School, New Brunswick, NJ, United States

**Keywords:** subarachnoid hemorrhage, microbiome, neuroinflammation, aneurysm, diet, intracranial hemorrhage

## Abstract

Subarachnoid hemorrhage (SAH) is a type of hemorrhagic stroke characterized by high morbidity and mortality. Saccular intracranial aneurysms account for most cases of SAH. While the role of hemodynamic stress and inflammation have been extensively studied in SAH, little is known about the role of the microbiome in SAH despite recent studies uncovering new insights on the effects of microbiome alteration in ischemic stroke. This review presents the current knowledge around the role of the microbiome in intracranial aneurysm formation and rupture. We also highlight the influence of diet on intracranial aneurysm formation and provide evidence that corroborates the targeting of inflammatory pathways as a potential strategy to curb SAH-associated neurological dysfunction.

## Introduction

Subarachnoid hemorrhage (SAH) is a life-threatening condition that results from the build-up of blood between the arachnoid and pia mater. SAH affects 10 to 14 out of 100,000 persons annually in the United States and is responsible for 10% of hemorrhagic strokes (de Rooij et al., 2007; Ziu, 2022). The etiology of SAH can be non-traumatic or traumatic. Most (85%) non-traumatic SAH arise secondary to intracranial aneurysm rupture (Ikawa et al., 2020). Risk factors such as age (>60), substance abuse (e.g., cocaine), smoking, hypertension, and genetic conditions have been shown to increase the risk of SAH and aneurysm rupture (Vlak et al., 2012).

Hemodynamic stress is a crucial factor responsible for aneurysm formation, especially at arterial junctions and bifurcations. Several studies have proposed that the hemodynamic stress on the arterial wall initiates inflammatory processes that significantly weaken the arterial wall and ultimately lead to rupture (Ziu, 2022). While several cytokines (e.g., interleukin-6) and metalloproteinases (MMPs) have been extensively studied for their role in intracranial aneurysmal formation and rupture (Sathyan et al., 2015; Zhang et al., 2019; Kaminska et al., 2022; Lucke-Wold et al., 2022), the role of the microbiome in aneurysm formation and rupture has only been appreciated in the past 3 years. However, the mechanisms through which the microbiome impact aneurysm formation and rupture remain unclear.

In this mini-review, we explore new insights into the effects of the microbiome on intracranial aneurysm formation and rupture. Since diet has been shown to influence the composition of the microbiota (Leeming et al., 2019), we examined studies demonstrating the influence of diet on intracranial aneurysm formation. Finally, we discuss relevant therapeutic targets of inflammation associated with intracranial aneurysm formation and rupture.

### A role for the microbiota-gut-brain axis in cerebrovascular hemorrhage

Over the last decade, our knowledge and understanding of the human microbiome and its link to various systems and pathologies has increased. The microbiota-gut-brain (MGB) axis is comprised of an intricate system of neural and immunological pathways with signaling molecules such as cytokines, hormones, neuropeptides, and bacterial metabolites that are believed to be used as communication between the microbiota, gut, and brain (Arpaia et al., 2013; Benakis et al., 2016; Chen et al., 2017). In particular, the MGB axis is thought to contribute to stroke outcome, as disruption of the gut vascular barrier (GVB) results in increased intestinal permeability and enables gut microbes to enter the bloodstream in animal models (Crapser et al., 2016; Chen et al., 2019; Lee et al., 2020). There is a paucity of literature concerning how the microbiome may influence intracranial aneurysm formation and rupture; thus, in this section, we briefly discuss recent findings demonstrating the role of the microbiome in several brain hemorrhagic conditions.

It has been suggested that commensal gut microbiota protects against ischemic stroke pathology and that their depletion leads to increased mortality and poor outcome, including worse neurological function (Singh et al., 2016; Winek et al., 2016). These findings become relevant given that SAH is a type of hemorrhagic stroke and has also been shown to cause delayed ischemic stroke (Martin and Rymer, 2011; Guresir et al., 2022). Compared to ischemic stroke not secondary to SAH, less is known about the association between SAH and the gut microbiome. A proof-of-concept study showed that antibiotic (vancomycin, metronidazole, ampicillin, and neomycin)-depletion of the gut microbiota in mice reduced the formation of intracranial aneurysms and neuroinflammation (Shikata et al., 2019). However, a significant limitation of the Shikata et al. (2019) study is the lack of clarity on which microbes are beneficial or harmful to aneurysm formation, given that the depletion of the gut microbiota with antibiotics represents a highly artificial scenario. Likewise, non-specific antimicrobial targeting of the gut microbiome may do more harm than good (Tang et al., 2017).

Two recent studies have addressed the contributions of specific microbes with regard to intracranial aneurysm formation and rupture. Notably, a prospective case-control microbiome study comparing the microbiome profile of patients with unruptured intracranial aneurysms (UIA) and ruptured intracranial aneurysms (RIA) demonstrated a significant increase in the relative abundance of *Campylobacter* at the phylum level in the RIA group compared to the UIA group. Further classification *via* 16S rRNA polymerase chain reaction at the genus level revealed that *Campylobacter* and *Campylobacter ureolyticus* were increased in the RIA group compared to the UIA group (Kawabata et al., 2022). Despite the intriguing findings from the Kawabata et al. study, some limitations exist. First, the composition of the gut microbiome prior to aneurysm rupture was not assessed. Second, the study did not collect patient dietary information, which could influence the gut microbiome. Finally, the study was conducted in a single country.

Microbes such as *Hungatella hathewayi* have also been implicated in intracranial aneurysm formation and rupture. In a case-control metagenome-wide associated study, Li et al. demonstrated that fecal transplant from UIA and control human patients to mice recipients was sufficient to induce UIAs in mice. The authors revealed that *Hungatella hathewayi* abundance was decreased in both human and mice UIAs and that the decrease in *Hungatella hathewayi* correlated to low levels of taurine, an anti-inflammatory metabolite known to be protective in stroke, traumatic brain injury, and SAH. Additionally, mice gavaged with *Hungatella hathewayi* were protected against intracranial formation and rupture (Menzie et al., 2013; Su et al., 2014; Jin et al., 2018; Li et al., 2020). While the Li et al. (2020) study demonstrated a direct causal effect of microbes on intracranial aneurysm formation and rupture, the study was performed in a single country, and it is unclear how *Hungatella hathewayi* works in concert with other microbes in decreasing aneurysm formation and rupture *via* modulation of taurine levels. Table 1 briefly summarizes the findings from the aforementioned SAH studies.

**Table 1:** Influence of the microbiome on intracranial aneurysm formation and rupture.

**SAH studies**	**Outcome**	**Proposed mechanism**	**References**
Antibiotic cocktail treatment	Depletion of the gut microbiota in mice reduced the formation of intracranial aneurysms and neuroinflammation	Macrophage infiltration and inflammatory cytokines disrupt the cerebral artery architecture	Shikata et al., 2019
Fecal Samples from UIA and RIA patients	*Campylobacter* and *Campylobacter ureolyticus* were increased in the RIA group compared to the UIA group	*Campylobacter* promotes vascular remodeling and cell death of cerebral artery walls *via* an inflammatory response and increased levels of matrix metalloproteinases	Kawabata et al., 2022
Fecal transplant from UIA and control human patients to mice	*Hungatella hathewayi* abundance was decreased in both human and mice UIAs Fecal transplant from human UIA to mice recipients was sufficient to induce UIAs in mice *Hungatella hathewayi* normalizes taurine levels Taurine supplementation reverses the progression of intracranial aneurysms	*Hungatella hathewayi* normalizes taurine, an anti-inflammatory metabolite that blunts inflammatory processes and ECM remodeling	Li et al., 2020

ECM, extracellular matrix; RIA, ruptured intracranial aneurysms; SAH, subarachnoid hemorrhage; UIA, unruptured intracranial aneurysms.

Like SAH, recent studies have suggested a role for microbes in intracerebral hemorrhage (ICH). A preclinical study observed that members of *Nocardiaceae, Helicobacteraceae, Veillonellaceae, Bacteroidaceae*, and *Akkermansiaceae* significantly increased ICH, while *Firmicutes, Barnesiellaceae, Bacteriidales*, and *Moraxellaceae* were reduced (Yu et al., 2021). Another study showed that compared to the sham group, experimental ICH depleted members of *Firmicutes*, such as *Faecalibaculum* and *Dubosiella*. In contrast, the members of *Proteobacteria* and *Campilobacterota*, such as *Enterobacter* and *Helicobacter*, were enriched (Xiao et al., 2022). Analysis of murine ileum tissue revealed the phylum *Proteobacteria*, the class *Gammaproteobacteria*, the order *Enterobacteriales*, the family *Enterobacteriaceae*, and the species *Escherichia coli* were higher in the gut microbiota early (1 day) following ICH, while by day 3 had a greater abundance of the family *Peptostreptococcaceae* and genus *Romboutsia* (Zhang et al., 2021). Furthermore, higher levels of gut microbiota metabolite short-chain fatty acids (SCFAs) and equol have been associated with a better outcome. In comparison, higher levels of trimethylamine-N-oxide (TMAO) have been associated with worse outcomes in cardiovascular disease, cerebrovascular disease, and intracranial aneurysm/hemorrhage (Wang et al., 2011; Tonomura and Gyanwali, 2021; Yokosuka et al., 2021; Zhai et al., 2021).

Many simultaneous pathways likely contribute to the interplay between hemorrhagic brain conditions and the MGB axis; these are considerable targets for therapeutic intervention. One such pathway is the NOD-like receptor family, pyrin domain-containing 3 (NLRP3) inflammasome. Administration of the selective NLRP3 inflammasome inhibitor, MCC950, modulated gut microbiota dysbiosis, increased the abundance of *Bacteroides, Bifidobacterium*, and *Paenibacillus*, and attenuated corticospinal tract injury and neurological deficits following experimental ICH (Xiao et al., 2022). Additionally, increased intestinal permeability was observed in mice with ICH compared to sham, and ICH mice exhibited worse intestinal motility and pathology (Yu et al., 2021; Zhang et al., 2021). Another potential pathway is the disruption of T-cell homeostasis. Yu et al. demonstrated T-cell accumulation in the brain following ICH, some of which had migrated from the intestine to the peri-hematoma region. Importantly, the recolonization of ICH mice with healthy microbiota through a fecal microbiota transplant ameliorates these deficits (Yu et al., 2021).

Cavernous angiomas (CAs), common vascular anomalies predisposing to brain hemorrhage characterized by dysmorphic dilated vascular capillaries lined by endothelium, have also been shown to affect the gut microbiome. A recent study showed that CA patients' microbiota were significantly enriched in lipopolysaccharide (LPS)-producing Gram-negative bacteria. This study identified five significantly contributive taxa, *Bifidobacterium adolescentis, Bacteroides eggerthii, Bacteroides dorei, Dorea*, and *Escherichia coli*, to distinguish aggressive from non-aggressive CA patients. Meanwhile, six taxa, including *Faecalibacterium prausnitzii, Oscillobacter, Lactobacillus rhamnosus, Enterobacter cloacae, Odoribacter laneus*, and *Bacteroides cellulosilyticus* were identified to be unique microbiome signatures of CA patients with symptomatic hemorrhage (Polster et al., 2020).

Despite studies providing evidence that microbial by-products modulate vascular inflammation; and thus contribute to the formation or rupture of intracranial aneurysms. It is unclear whether the direct transmigration of gut microbe into cerebral vessels represents a potential mechanism responsible for intracranial aneurysm formation and rupture. Furthermore, future longitudinal human studies are needed to assess the microbiome composition in patients at high risk for intracranial aneurysms, as this could provide insight into understanding the cause-effect relationship between the gut microbiome and intracranial aneurysms.

### The influence of diet on aneurysm formation and rupture

A myriad of dietary metabolites influence the risk of SAH formation and rupture. Inadequate intake of dietary antioxidants, hyperhomocysteinemia, hypertension, and alcohol consumption may increase the risk of intracranial aneurysms. At-risk individuals should consider diets that provide sufficient amounts of antioxidant vitamins, B vitamins, flavonoids, and n-3 fatty acids while limiting alcohol and caffeine consumption. Healthy diets such as the Mediterranean diet or Dietary Approach to Stop Hypertension (DASH) diet promote high consumption of vegetables, fruits, legumes, whole grains, nuts, fish, and herbs, which provide those necessary nutrients listed above, and are thus recommended for the prevention of intracranial aneurysms (Czekajlo, 2019; Cao et al., 2021). Interestingly, adherence to a DASH diet was inversely associated with the risk of ischemic stroke and ICH, but not SAH (Larsson et al., 2016).

Vitamin E supplementation in cholesterol-treated rabbits decreased the degree of vasospasm following basilar artery SAH (Sasani et al., 2011). This is contrary to an earlier clinical trial, which showed that vitamin E (*dl-alpha-tocopherol*) supplementation in hypertensive men decreased the risk of cerebral infarction but increased the risk of SAH; however, this effect was observed in hypertensive subjects (Leppala et al., 2000). Given this disparity in results, future clinical trials are needed to elucidate the role of vitamin E in SAH pathophysiology. Polyphenols, plant secondary metabolites in foods such as fruits, vegetables, beverages including tea and red wine, and extra virgin oil, are beneficial in stroke (Parrella et al., 2020). Cadmium, a toxic metal associated with several adverse health effects, exposure occurs mainly from the diet (whole grain rice and potatoes) and tobacco smoke. Cadmium's excretion is very slow, with a half-life of decades, and this measure has been positively associated with stroke, myocardial infarction, and abdominal aortic aneurysm. Cadmium levels measured in the blood of the Malmo Diet and Cancer cohort (93 SAH cases vs. 276 matched controls) revealed patients with the highest cadmium levels had an increased risk of SAH, which was primarily due to smoking (Soderholm et al., 2020). A major limitation of the Soderholm et al. (2020) study was the low number of SAH cases. Although a few case reports have implicated the use of dietary supplements such as *ginkgo biloba* and spontaneous cerebral hemorrhage (e.g., ICH, SAH, and SDH), more studies are needed to fully establish this risk (Rosenblatt and Mindel, 1997; Benjamin et al., 2001; Friedman et al., 2007; Stanger et al., 2012).

High-fat diet in rats promotes increased serum cholesterol and facilitates enlargement and degenerative changes in the media of intracranial aneurysms, presumably through foam cell transformation (Shimizu et al., 2019). The population-based case-control Australasian Cooperative Research on Subarachnoid Hemorrhage Study (ACROSS), consisting of 432 first-ever cases of SAH vs. 473 controls, found frequent fat intake was associated with SAH risk. In contrast, frequent skim or reduced-fat milk and fruit intake were protective against SAH (Shiue et al., 2011). In comparison, the Japan Public Health Center-based prospective (JPHC) Study found that dietary saturated fatty acids (SFAs) were inversely associated with deep intraparenchymal hemorrhage and lacunar infarction but had no association with SAH (Yamagishi et al., 2013). Interestingly, pretreatment with omega-3 fatty acids protected against SAH in rats *via* the G protein-coupled receptor 120/β-arrestin2/TGF-β activated kinase-1 binding protein-1 anti-inflammatory signaling pathway, suggesting that fish oil supplementation as part of a daily diet may contribute to improved clinical outcomes in SAH patients (Yin et al., 2016).

The European Prospective Investigation into Cancer and Nutrition (EPIC)-Oxford cohort showed that while fish eaters and vegetarians had lower rates of ischemic heart disease, vegetarians had higher rates of hemorrhagic and total stroke (i.e., ischemic and hemorrhagic stroke) (Tong et al., 2019). Data from two prospective population-based cohorts of Swedish women and men showed that while total fruit and vegetable consumption was significantly inversely associated with total stroke, SAH alone was not (Larsson et al., 2013). Another study from the Danish Diet, Cancer, and Health cohort found that adherence to the EAT-Lancet diet, a sustainable, and primarily plant-based diet, was associated with a lower risk of SAH, while the Alternate Healthy Eating Index-2010 (AHEI) was associated with a lower risk of ischemic stroke (Ibsen et al., 2022).

While several population studies have demonstrated the role of diet in the pathogenesis of intracranial aneurysms, it remains unclear how diet indirectly alters the gut microbiome composition in a way that could promote or mitigate intracranial aneurysm formation. Likewise, future studies examining mechanistic pathways on how specific diet components promote or mitigate intracranial aneurysm formation are needed.

### Neuroinflammation as a therapeutic target in subarachnoid hemorrhage

The neuroinflammatory response to acute central nervous system (CNS) insult involves a multimodal activation of peripheral and central immune cells and mediators. This inflammatory cascade is a critical pathologic force in the development and progression of intracranial aneurysms, in addition to acute and chronic consequences of SAH. Furthermore, recent studies suggest that gut microbes promote intracranial aneurysm formation and rupture *via* modulation of vascular inflammation; thus, targeting vascular inflammation may be therapeutically beneficial in SAH management (Shikata et al., 2019; Li et al., 2020; Kawabata et al., 2022).

### Inflammatory factors in the development, growth, and rupture of intracranial aneurysms

Endothelial injury, a core process in aneurysm pathogenesis, invokes a coordinated inflammatory response, first through increased proinflammatory (i.e., COX2-PGE_2_-EP_2_-NF_k_B) signaling and subsequently through the infiltration of inflammatory cells (Aoki et al., 2011; Chalouhi et al., 2012). The resultant “inflammatory zone,” consisting of macrophages, T-cells, mast cells, and cytokines, causes vascular smooth muscle cells (vSMCs) within the media to adopt a proinflammatory matrix remodeling phenotype. TNF-alpha contributes to the reprogramming of normal contractile vSMCs to a pathologic phenotype resulting in myointimal hyperplasia (Kosierkiewicz et al., 1994; Ali et al., 2013). These vSMCs exhibit reduced expression of contractile proteins and production of inflammatory factors and matrix metalloproteases (MMPs) (Starke et al., 2014). MMPs produced by vSMCs and macrophages incite damage against the vessel wall, disrupting the internal elastic lamina (IEL) and extracellular matrix (Texakalidis et al., 2019). Disruption of the IEL by this inflammatory cascade appears to be a critical event in aneurysm formation (Kim et al., 1993). The aneurysm's continued growth depends on a complex, coordinated degenerative and remodeling process (Frosen et al., 2019). Rupture results from the continued weakening of the wall, specifically the replacement of collagen and muscular layers with a hyaline-like substance (Kataoka et al., 1999). Furthermore, it appears that a shift in vSMCs from the proinflammatory phenotype to apoptosis and subsequent thinning of the media is a late event preceding aneurysm rupture (Guo et al., 2007).

### Acute and sub-acute neuroinflammatory response to subarachnoid hemorrhage

Inflammation, edema, ischemia, and cell death, are the primary pathologic processes underlying early brain injury (0–72 h) following subarachnoid hemorrhage. The initial rapid accumulation of blood in the subarachnoid space—most often resulting from aneurysmal rupture—results in an immediate rise in intracranial pressure and a corresponding reduction in cerebral blood flow (Conzen et al., 2019). The consequent parenchymal metabolic dysfunction and neuronal apoptosis/necrosis incite microglia, the resident immune cells of the CNS, to become activated in regions closest to the bleeding (Provencio et al., 2011). The binding of pathogen- or damage-associated molecular patterns (PAMPs/DAMPs), such as heme from lysed erythrocytes to Toll-like receptors (TLRs), including TLR2 and TLR4, is implicated as the cellular mechanism underlying microglial activation (Hanafy, 2013; Xu et al., 2021; Islam et al., 2022). During the acute period, microglia are thought to adopt an M1 polarization characterized by neurotoxicity (Li et al., 2018). This drives further neuronal death, blood-brain barrier permeability, and recruitment of peripheral inflammatory cells (Schneider et al., 2015). Infiltration of neutrophils, the most abundant of the circulating leukocytes, is considered a hallmark of post-SAH inflammation which drives early tissue damage and neuronal death. Indeed, neutrophil accumulation within the cerebral vasculature and parenchyma has been identified as early as 10 min following hemorrhage and peaks between 3–4 days following (Friedrich et al., 2011; Atangana et al., 2017). Experimental preclinical studies utilizing the depletion of neutrophils have determined that early infiltration is responsible for microvascular injury and resultant local ischemia, as well as aggravation of the inflammatory response and neuronal death (Friedrich et al., 2011; Atangana et al., 2017; Neulen et al., 2019).

### Delayed neuroinflammatory response to subarachnoid hemorrhage

Persistent neuroinflammatory changes following subarachnoid hemorrhage have been identified; however, little is known about their pathophysiologic implications. Notably, microglial polarization appears to shift from the proinflammatory, neurotoxic (M1) to anti-inflammatory, neuroprotective (M2) phenotype. TNF-stimulated gene-6 (TSG-6), a glycoprotein that comprises the negative feedback loop which tempers inflammatory progression, has emerged as one molecular mediator of this transition (Li et al., 2018). Vasospasm and cerebral ischemia are clinical hallmarks of the delayed period following subarachnoid hemorrhage and represent the primary cause of morbidity and mortality. Irritation of the cerebral vessels by blood breakdown products like hemoglobin has been the classical hypothesis of delayed cerebral ischemia. However, the failure of agents targeting vasospasm to mitigate neurological deterioration suggests that microvascular injury, thrombosis, and inflammation may be more pervasive processes (Dodd et al., 2021). Indeed, monoclonal antibody-mediated neutralization of secreted HMGB1 was shown to attenuate microglial activity, ameliorate the vasocontractile response to thrombin, and improve locomotor recovery (Haruma et al., 2016). Consistent with this, elevated HMGB1 in cerebrospinal fluid correlated with levels of inflammatory cytokines and was associated with unfavorable neurological outcomes (Nakahara et al., 2009). These specific examples support the generally well-appreciated relationship between heightened systemic immune response, delayed ischemia, and poor neurological outcome (Al-Mufti et al., 2019; Ma et al., 2021).

### Considerations for therapeutic targeting of neuroinflammation in subarachnoid hemorrhage

The pervasiveness of inflammatory processes in multiple phases of subarachnoid hemorrhage pathophysiology renders them clear targets for future therapeutics. However, successful targeting will depend on coordinating the cellular target and appropriate timing of intervention. For example, the administration of pharmacological agents that favor the anti-inflammatory (M2) phenotype early after hemorrhage may moderate microglial activation and polarization (Schneider et al., 2015). Additionally, the unintended effects of manipulating the inflammatory response must be considered. While induction of neutropenia mitigates peripheral inflammatory infiltration and subsequent microvascular injury, agents used to induce the neutropenic state can predispose to systemic infection or impair coagulative responses (Friedrich et al., 2011). This is borne out in clinical literature where utilization of corticosteroids to modulate inflammation has demonstrated no benefit at best and potential risk for harm (i.e., infection, hyperglycemia) at worst (Feigin et al., 2005; Czorlich et al., 2017).

## Conclusion

**Figure 1:**
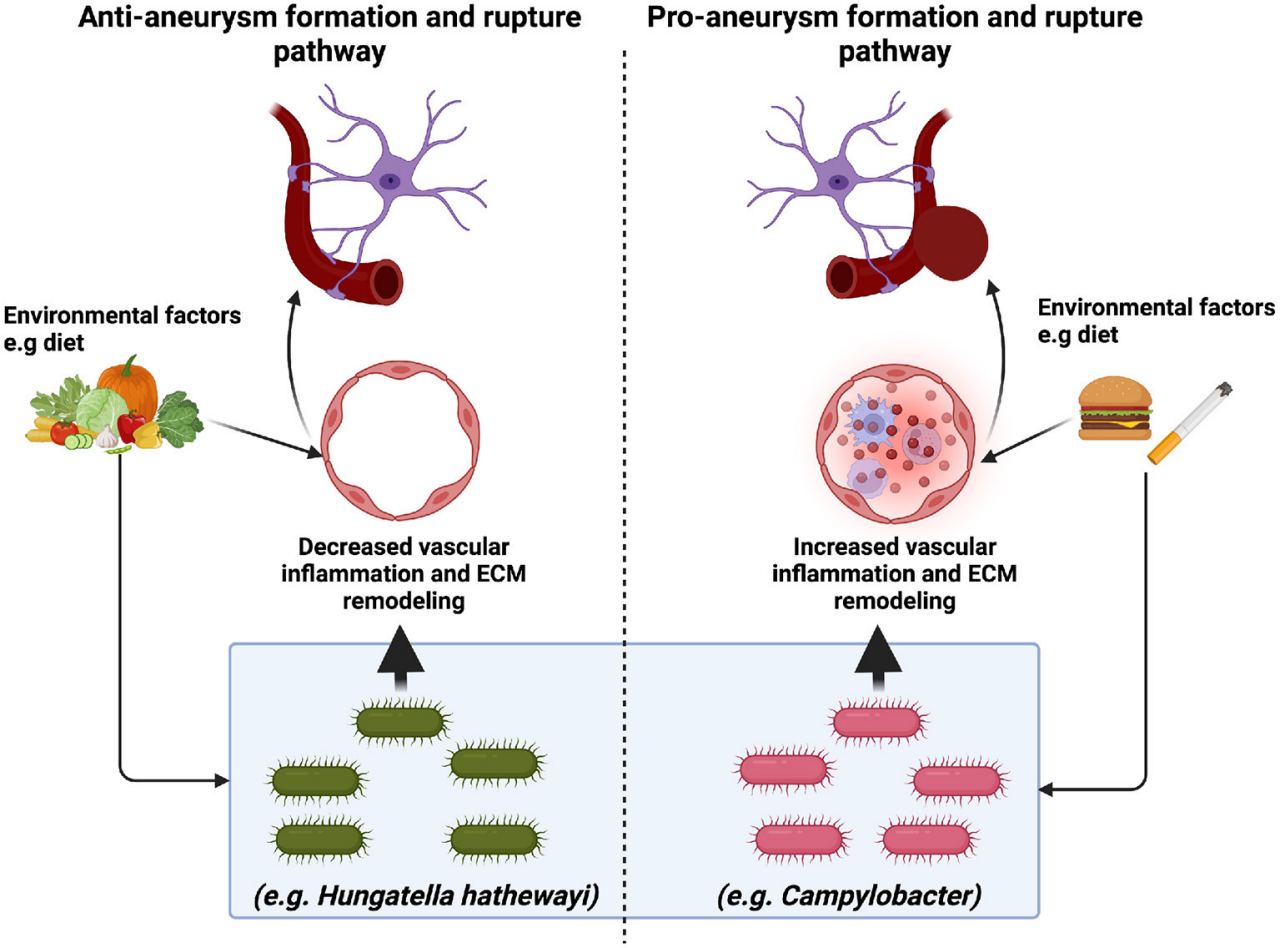
The implications of the microbiome in intracranial aneurysm formation and rupture. Recent studies suggest that specific microbes may promote (*Campylobacter*) or mitigate (*H. hathewayi*) intracranial aneurysm formation and rupture. Pro-aneurysm microbes are thought to induce vascular inflammation (immune cell infiltration and extracellular matrix (ECM) remodeling). In contrast, anti-aneurysm microbes are thought to decrease vascular inflammation *via* anti-inflammatory metabolites such as taurine. While it is clear that environmental factors such as diet may alter the gut microbiome, the mechanism through which this occurs is unclear. Future studies are needed to elucidate further the role of diet and the gut microbiome in intracranial aneurysm formation and rupture.

While we broadly understand the role of inflammatory cytokines and other risk factors (e.g., hypertension, smoking, etc.) on intracranial aneurysm formation and rupture, further insights on the role of the microbiome and its interplay in the pathways as mentioned above with regards to intracranial aneurysm formation and rupture remains to be elucidated. The recent microbiome studies discussed in this review and the findings summarized in Figure 1 emphasize the importance of the gut microbiome intracranial formation and rupture. Further studies are needed to delineate the effects of specific microbes on intracranial aneurysm formation and rupture. Likewise, a functional understanding of how microbes work in concert to modulate intracranial aneurysm formation and rupture is needed. These studies will rely heavily on developing new preclinical models and tools that accurately resemble the human clinical progression of intracranial aneurysm rupture and allow for the detection of aneurysms prior to euthanasia.

## Author contributions

All authors listed have made a substantial, direct, and intellectual contribution to the work and approved it for publication.
